# Review of the correlation between Chinese medicine and intestinal microbiota on the efficacy of diabetes mellitus

**DOI:** 10.3389/fendo.2022.1085092

**Published:** 2023-01-25

**Authors:** Min Su, Rao Hu, Ting Tang, Weiwei Tang, Chunxia Huang

**Affiliations:** ^1^ Hunan Key Laboratory of The Research and Development of Novel Pharmaceutical Preparation, Changsha Medical University, Changsha, China; ^2^ Department of Biochemistry and Molecular Biology, School of Basic Medicine, Changsha Medical University, Changsha, China

**Keywords:** traditional Chinese medicine, diabetes, metabolism, active ingredients, intestinal microbiota

## Abstract

Diabetes mellitus is a serious metabolic disorder that can lead to a number of life-threatening complications. Studies have shown that intestinal microbiota is closely related to the development of diabetes, making it a potential target for the treatment of diabetes. In recent years, research on the active ingredients of traditional Chinese medicine (TCM), TCM compounds, and prepared Chinese medicines to regulate intestinal microbiota and improve the symptoms of diabetes mellitus is very extensive. We focus on the research progress of TCM active ingredients, herbal compounds, and prepared Chinese medicines in the treatment of diabetes mellitus in this paper. When diabetes occurs, changes in the abundance and function of the intestinal microbiota disrupt the intestinal environment by disrupting the intestinal barrier and fermentation. TCM and its components can increase the abundance of beneficial bacteria while decreasing the abundance of harmful bacteria, regulate the concentration of microbial metabolites, improve insulin sensitivity, regulate lipid metabolism and blood glucose, and reduce inflammation. TCM can be converted into active substances with pharmacological effects by intestinal microbiota, and these active substances can reverse intestinal microecological disorders and improve diabetes symptoms. This can be used as a reference for diabetes prevention and treatment.

## 1 Introduction

Diabetes mellitus [Xiaokezheng or Xiaodanzheng in traditional Chinese medicine (TCM)] is a serious disorder of metabolism to islets. Insulin resistance (IR) and impaired islet cell functions are characterized by an increase in glycemia, lipid metabolism disorders, and systemic inflammation that can lead to a variety of serious complications (1). According to the World Health Organization, diabetes is one of the diseases with the most known complications (2, 3). Long-term increases in blood glucose are accompanied by large blood vessels and microvascular damage, putting the heart, brain, kidneys, peripheral nerves, eyes, feet, and other organs at risk. Type 2 diabetes mellitus (T2DM) is one of the world’s most significant public health issues, accounting for 90% of diabetes cases (4). T2DM has been rapidly increasing worldwide in recent years (5).

An important internal environment for human physiological and metabolic activities is a healthy intestinal environment. More and more studies indicate that changes in the gut microbiota are associated with altered glucose homeostasis and play an important role in the onset and progression of obesity and T2DM. The host’s intestinal microbiota influences body weight, bile acid metabolism, proinflammatory activity, IR, and gut hormone modulation (6). Reduced microbial diversity has been linked to IR and energy metabolism, particularly as the *Firmicutes/Bacteroidetes* (F/B) ratio rises (7). The variation in fecal microbiome trends between normal and obese T2DM patients was different (8, 9). For example, Liu Shixuan et al. compared the abundance of intestinal microbiota in diabetic patients and controls, finding that *Actinomycetes*, *Clostridium*, *Escherichia*, and *Proteus* were significantly enriched in the T2DM group, while *Rossella*, *Eubacterium*, and *Faecalibacterium* decreased (10). *Roseburia*, *Eubacterium*, and *Faecalibacterium* are all members of the phylum Pachylocycetes and have been shown to promote intestinal barrier repair and inhibit inflammatory factors (11). It is clear that changes in this microbiota will have an impact on the intestinal barrier function. The reason is the change of proteins related to the maintenance of the intestinal barrier.

Diabetes treatment options are numerous at the moment. However, the effect of a drug on the intestinal microbiota is a very important evaluation index for drug safety and efficacy. TCM has shown remarkable efficacy in the treatment of diabetes. In practice, Chinese medicine has summed up many classic prescriptions for the treatment of diabetes (12, 13). At present, there are extensive studies on the active ingredients of TCM, TCM compounds, and prepared Chinese medicines to regulate the structure of the intestinal microbiota and improve the symptoms of diabetes. So many studies have shown that there is an interactive relationship between intestinal microbiota and TCM. On one hand, intestinal microbiota can transform the active ingredients in herbal medicine, and on the other hand, these active ingredients can reverse the imbalance of intestinal microbiota, and the symptoms of diabetes can be improved after the intestinal microbiota imbalance is restored (14–17). Therefore, the gut microbiota can be used as a target for TCM to prevent and treat T2DM (18). This review aims to provide the relationship between T2DM, intestinal microbiota, and TCM, as well as the mechanisms of action of herbal components in metabolic diseases. This could help future clinical trials of TCM for the treatment of diabetes mellitus.

## 2 Correlation of intestinal microbiota with T2DM and its mechanism of action

**Figure 1:**
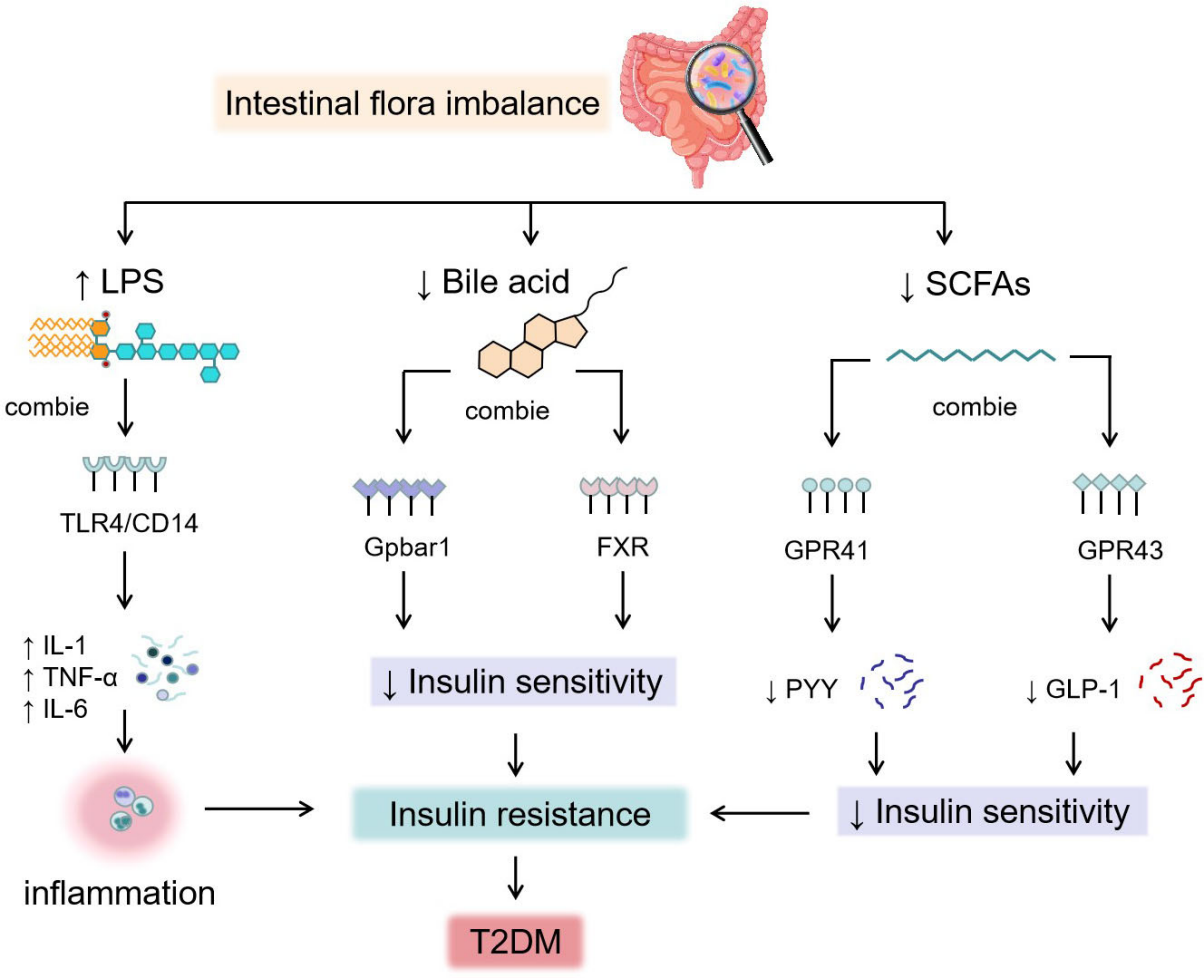
The mechanism of action of the intestinal microbiota and diabetes.

The composition of intestinal bacteria is more diverse in healthy people, whereas obese or diabetic patients have a reduced diversity of intestinal microbiota, with more conditionally pathogenic bacteria and less beneficial microbiota. The intestinal microbiota and its metabolites travel from multiple pathways involving the body’s physiological processes, including immunomodulation, metabolism, and even brain function (19). In the studies of the “microbial-gut-brain-hepatic axis”, the intestinal microbiota controls intestinal enteroendocrine cells (EECs); they could secrete cholecystokinin (CCK), leptin, peptide YY (PYY), glucagon-like peptide-1 (GLP-1) and 5-hydroxytryptophan (5-HT). These intestinal peptides could cause the disorders of glucose and lipid metabolism by regulating the central nervous system and associated signaling pathways (20). *Clostridium*, *Bacillus*, *Enterococcus*, *Bifidobacterium*, *Lactobacillus*, and *Bacteroides* could produce bile salt hydrolase (BSH). BSH could act on amino acids that conjugate bile acids, produce free bile acids, and further modify the formation of secondary bile acids. Bile acids combined with the G protein–coupled bile acid receptor (Gpbar1) and farnidol X receptor (FXR) regulates glucose and lipid metabolism, stimulates the release of PYY and GLP-1, increases the body’s sensitivity to insulin, and reduce the glycemia levels (21–25). *Vibrio butyrates*, *Rochesella*, *faecochoncilli*, *Bifidobacterium*, *Eubacterium* and *Clostridium flex* can cause fermentation and produce short-chain fatty acids (SCFAs). SCFAs combined with G protein-couple receptor (Gpr41/Gpr43) can also stimulate the release of PYY and GLP-1, improving IR. Dysbacteriosis in T2DM patients leads to an increase in Gram-negative bacteria–producing lipopolysaccharides (LPSs) and a decrease in the microbiota that protect the intestinal mucosal barrier (26). Thus, the expression of intestinal epithelial tight-junction proteins is inhibited, and the permeability of the intestine increases, promoting the absorption of LPSs (27). LPSs activate receptors (CD14/TLR4) on the surface of immune cells; promote interleukin-1 (IL-1), tumor necrosis factor-α (TNF-α), interleukin-6 (IL-6), and other proinflammatory factor secretions; and induce chronic low-grade inflammation, and long-term accumulation weakens the body’s responsiveness to insulin and induces IR (28). The mechanism of action of the intestinal microbiota and diabetes is shown in **Figure 1**.

Probiotics have been reported to modulate the intestinal microbiota to prevent or delay the onset of T2DM by including improving the gut barrier, improving intestinal integrity, alleviating inflammation, increasing glucagon-like peptide (GLP) 1 and GLP 2, increasing the production of SCFAs, decreasing LPS levels and endoplasmic reticulum stress, improving peripheral insulin sensitivity, and so on (29). The above proves that adjusting the gut microbiota can treat diabetes.

## 3 Effects of Chinese herb ingredients on intestinal microbiota

A review has shown that the abundance of bioactive constituents in Chinese herbs has a protective effect on the balance of the intestinal microecosystem, directly or indirectly regulating the imbalance of the intestinal microbiota (30). The possible mechanisms by which the active ingredient of Chinese herbs intervenes in the intestinal microbiota in the treatment of T2DM are as follows.

### 3.1 Polysaccharides

Polysaccharides are a class of natural polymers made of aldose or ketose connected by glycoside bonds, which are commonly found in botanicals. Chinese herbal polysaccharides improve glycolipid metabolism, inflammation, and intestinal barrier regulation, primarily by regulating intestinal microbiota species. Lycium barbarum polysaccharides (LBPs) have been reported to ameliorate diabetes by enhancing the gut barrier *via* modulating the gut microbiota and activating gut mucosal TLR2+ intraepithelial γδ T cells in rats. LBP could suppress inflammation in T2DM by decreasing the levels of plasma proinflammatory IL-1β, IL-6, IL-17A, and TNF-α in diabetic rats. LBP also could reduce the levels of glycated hemoglobin (GHb), triacylglycerol (TG), and total cholesterol (TC) (31). A polysaccharide isolated from Ganoderma lucidum ameliorates hyperglycemia by repairing islet cells and increasing insulin secretion, promoting the synthesis and storage of glycogen in the liver and improving the activities of antioxidant enzymes and IR (32). Moutan cortex polysaccharides also reconstruct gut microbiota, improve intestinal barrier function, reduce serum proinflammatory mediators, and elevate the SCFA contents. Moutan cortex polysaccharides alleviate diabetic kidney disease (DKD) in rats (33). Cyclocarya paliurus polysaccharides modulate the gut microbiota and SCFAs by stimulating SCFA receptors including GPR41, GPR43, and GPR109a and upregulating the expression of GLP-1 and PYY, thereby treating alleviate T2DM symptoms (34).

### 3.2 Saponins

Saponins are widely found in licorice, bupleurum, ginseng, Panax notoginseng, astragalus, and other Chinese herbs that play an important role in the prevention and treatment of diabetes. Polygonatum sibiricum saponin could decrease the abundance of Firmicutes in T2DM rats and increase the abundance of Bacteroidetes; thereby, it could significantly decrease the levels of insulin secretion and fasting blood glucose (FBG), TG, TC, and low-density lipoprotein cholesterol (LDL-C) and increase the content of high-density lipoprotein cholesterol (HDL-C) (35). Ginsenoside Rg1 treatment improves blood glucose, the blood lipid profile (total cholesterol, triglycerides, and LDL-C levels), the IR index, and liver function (aspartate transaminase and alanine transaminase levels). The levels of inflammatory cytokines (including IL-6, IL-1, and TNF-a) substantially decreased after ginsenoside Rg1 treatment (36). Other studies show that ginsenoside Rg5 improved the symptoms of hyperglycemia, repaired intestinal barrier function, relieved metabolic endotoxemia-related inflammation, and reversed gut microbiota dysbiosis in the colon with significantly decreased F/B ratios. More importantly, the effects of ginsenoside Rg5 were further confirmed by partial changes in the gut microbiota induced by broad-spectrum antibiotics because ginsenoside Rg5 increased the abundance of *Bacteroidetes* and *Proteobacteria* and dramatically decreased the abundance of *Firmicutes* and *Verrucomicrobia* in the gut of diabetic db/db mice (37). Oleuropein can increase the relative abundance of *Verrucomicrobia* and *Deferribacteres* and decreases the relative abundance of *Bacteroides.* Thereby, it has a significant effect on improving glucose tolerance, decreasing FBG levels, lowering the homeostasis model assessment–IR index, and improving diabetes-related metabolic disorders (38). Aloin modulated the bacterial community in the gut by raising the abundance of *Bacteroidota* and reducing the richness of *Firmicutes*, *Proteobacteria*, and *Actinobacteriota*. Thus, aloin ameliorated IR *via* activating the IRS1/PI3K/Akt signaling pathway and regulating the gut microbiota, Thereby, it diminished weight loss, reduced FBG levels and hemoglobin A1c activity, and promoted glucose tolerance and fasting serum insulin activity in T2DM rats (39).

### 3.3 Polyphenols

Polyphenols are secondary metabolites found abundantly in a wide variety of Chinese herbs. The polyphenols and intestinal microbiota could interact with each other. The polyphenols in Chinese herbs can be further transformed, absorbed, and utilized by the intestinal microbiota. At the same time, polyphenols can regulate the composition of the intestinal microbiota by inhibiting pathogenic bacteria and promoting the growth of beneficial bacteria. There are reports that oral treatment with 200 mg/kg honokiol for 8 weeks significantly decreases the FBG in T2DM rats. The phosphorylation of the phospho-insulin receptor B-subunit (IR) and downstream insulin signaling factors such as AKT and ERK1/2 increased in a dose-dependent manner in the adipose, skeletal muscle, and liver tissue of honokiol-treated rats. Honokiol may also improve insulin-stimulated GLUT4 translocation by increasing the abundance of *Akkermansia* and SCFA-producing *Bacteroides* while decreasing *Oscillospira* (40, 41). Curcumin significantly improved gut integrity, hyperglycemia, IR, and endotoxemia in diabetic rats by reversing intestinal microbiota disturbances in diabetic rats (42). Interestingly, resveratrol also lowered blood glucose levels by modulating the gut microbiota but not when combined with curcumin, which may be related to their differential regulation of the gut microbiota (43).

### 3.4 Alkaloids

Alkaloids are a class of nitrogen-containing organic compounds that are an important part of Chinese herbs and have anti-inflammatory, antidiabetic, anti-obesity, and antihyperlipidemic effects (44). Berberine was found to reduce the Bacteroidetes/Firmicutes ratio in a study. Berberine, through its direct antibacterial action, could regulate glucose metabolism and body weight in Goto–Kakizaki (GK) rats by inhibiting the growth of harmful bacteria. Analysis indicated that FBG was negatively correlated with *Allobaculum* and strongly positively correlated with *Clostridia*. Body weight showed a negative correlation with *Akkermansia* and a positive correlation with *Desulfovibrionaceae (* 45). Berberine action with intestinal bacteria reduced serum MCP-1, TNF-, and IL-6 expression, which was accompanied by decreased levels, improving glucose tolerance in diabetic rats (46). Total alkaloids of *Corydalis saxicola* (TACS) action caused the mouse intestinal microbiota to return to a controlled level. TACS could intervene in intestinal microbial disorders through four metabolic pathways (BCAAs), bile acids, arginine and proline, and purine metabolism (47). Mulberry total biotobasine significantly increased the abundance of microbiota that promote SCFA production such as *Erysipelotrichaceae* and *Bacteroides* and reduced the abundance of harmful microbiota such as *Desulfovibrio* and *Rikenellaceae*. Therefore, mulberry total biotobasine promotes insulin secretion and ameliorates the β cells of diabetes rats’ dysfunction and mass reduction both *in vivo* and *in vitro* (48).

### 3.5 Licorice extract

High-dose licorice extract could effectively decrease the levels of nuclear factor kappa-B (NF-κB), toll-like receptor 4 (TLR4) through reshaping the gut microbiota structure. At a normal glucose level, licorice extract decreased AMP-activated protein kinase α (AMPKα) phosphorylation, and at high glucose, licorice extract augmented the cytosolic calcium concentration. Therefore, it alleviated hyperglycemia and glucose intolerance in T2DM rats and improved the function and morphology of diabetic islets. More importantly, all the doses of licorice extract regulate intestinal microbiota balance by increasing the contents of *Akkermansia*, *Alloprevotella*, and *Bacteroides* and decreasing the contents of *Lachnospiraceae*, especially for the high dose of licorice extract. Licorice extract could also alleviate serum LPSs and FBG. These results indicated that the antidiabetic effect of licorice extract might be attributed to the regulation of the gut microbiota and the colon TLR4/NF-κB signaling pathway in diabetic rats (49).

### 3.6 Flavonoids

Pueraria, Scutellaria, Gingko leaves, and other medicines contain flavonoids, which have a wide range of biological activities. The gut microbiota play essential roles in the digestion and absorption of flavonoids and affect the occurrence and progression of T2DM. Flavonoids effectively increased insulin levels, decreased FBG content, reduced lipid accumulation in plasma, alleviated oxidative injury and inflammation, and relieved liver and kidney damage in diabetic mice (50). For example, Pueraria extract contains nine flavonoids; they can increase intestinal probiotics to improve metabolic disorders caused by diabetes and decrease *Clostridium celatum* levels to alleviate inflammation. Flavonoids have the potential to be used to control type 2 diabetes by regulating glycolipid metabolism and inflammation levels (50, 51).

### 3.7 Ethanol extract of Sargarsum fusiforme

The ethanol extract of Sargarsum fusiforme could significantly reduce food intake, water intake, and FBG while improving glucose tolerance, blood lipid levels, and hepatic oxidative stress in diabetic rats. The ethanol extract of Sargarsum fusiforme could reduce the abundance of bacteria related to diabetes or other metabolic diseases (such as *Romboutsia* and *Enterorhabdus*), and increase the abundance of benign bacteria (such as *Lachnoclostridium* and *Intestinimonas*). In the gut contents of diabetes rats, branched-chain amino acid levels were decreased, aromatic amino acid levels were decreased, and 4-hydroxyphenylacetic acid levels were increased, suggesting that EE may alter the ratio of these compounds by modulating the gut microbiota while affecting T2DM (52).

The mechanism of Chinese medicinal ingredients acting on intestinal microbiota in the treatment of diabetes is shown in **Table 1**.

**Table 1:** Progress on the mechanism of Chinese medicinal ingredients acting on intestinal microbiota in the treatment of diabetes.

TCM Ingredients	Effects on gut microbiota		metabolites		Therapeutic effect	Document
	Promotes	Reduces	Promotes	Reduces		
**Polysaccharide**	*Lactobacillus* *Bacteroides Ruminococcaceae* *Bifidobacterium* *Alistipes*	*Blautia Desulfovibrio*	SCFAs PYY GLP-1	GHb TG, TC LPS	Improves diabetes-related biochemical abnormalities and alleviates type 2 diabetes symptoms	34920067 35307460 32663709
**Saponins**	*Bacteroides* *Proteobacteria*	*Firmicutes* *Verrucomicrobia* *Proteobacteria* *Actinobacteriota*	HDL-C insulin	FBG TG, TC LDL	Improves insulin resistance and symptoms of hyperglycemia, repairs intestinal barrier function, relieves inflammation, and reverses gut microbiota dysbiosis	34517283 28362999 32307991 34206641 31181236
**Polyphenols**	*Akkermansia Bacteroides*	*Oscillospira* *Firmicutes*	SCFAs insulin	FBG	Improves the insulin sensitivity and reduces blood glucose	26674084 31921106 34818970 31473511
**Alkaloid**	*Erysipelotrichaceae* *Bacteroides*	*Desulfovibrio* *Rikenellaceae*	SCFAs	FBG LPS	Regulates blood glucose, improves blood lipids, and reduces insulin resistance	30837071 33716449 27702567 31214133 35308210
**Licorice extract**	*Akkermansia* *Bacteroides Alloprevotella*	*Lachnospiraceae*	*−*	FBG LPS TG, TC	Improves insulin resistance, serum lipids, and endotoxemia-related colonic inflammation.	35227470
**Pueraria leaf extract**	*Probiotics*	*Clostridium*	insulin	FBG	Ameliorates oxidative injury and inflammation and relieves liver and kidney damage	35289350
**Flavonoids**	*Akkermansia*	*Clostridium*	insulin	FBG TG, TC	Alleviates oxidative injury and inflammation and relieves liver and kidney damage	35289350
**Ethanol extract of Sargarsum fusiforme**	*Lachnoclostridium* *Intestinimonas* *Oscillibacter*	*Romboutsia Enterorhabdus* *Lachnospiraceae*	4-HA	FBG AAA BCAA	Improves the level of inflammation and reduces blood glucose.	34399527

## 4 Effects of Chinese herbal compounding on intestinal microbiota and diabetes

Chinese herbal compounding has been used to treat diabetes for thousands of years. These traditional remedies mostly contain herbs that benefit Qi. The main effects and their mechanisms are as follows.

### 4.1 Huang-Lian-Jie-Du decoction

It was discovered that Huang-Lian-Jie-Du decoction (HLJDD) could improve metabolic disturbances such as lipids, hyperglycemia, and inflammation; shape the microbiome; and restore dysregulated microbiota function in T2DM rats. HLJDD treatment could not only restore gut dysbiosis in T2DM rats, as evidenced by an increase in SCFA-producing and anti-inflammatory bacteria(e.g., *Akkermansia*, *Blautia*, and *Parabacteroides*), as well as a decrease in conditioned pathogenic bacteria (e.g., *Aerococcus*, *Corynebacterium*, and *Staphylococcus*), but also modulate the dysregulated function of the gut microbiome in T2DM rats (53). The changes in intestinal microbial populations may be due to the action of the active ingredients saponin and berberine in HLJDD.

### 4.2 Xie-xin decoction

The Xie-xin decoction (XXD) from Zhang Zhongjing’s Medical Treasures of the Golden Chamber is a compound recipe for heat-clearing and detoxication. Since the Tong Dynasty, this classic prescription of Dahuang, Huanglian, and Huangqin has been widely used to treat diabetes with remarkable therapeutic effects (54). According to one study, XXD can significantly improve hyperglycemia, lipid metabolism, and inflammation in T2DM rats by increasing the abundance of the gut microbiota, particularly some SCFA-producing and anti-inflammatory bacteria (e.g., *Alloprevotella*, *Adlercreutzia*, *Blautia*, *Barnesiella*, *Papillibacter*, and *Lachnospiraceae*) (55). The mechanism of action may be related to the various flavonoids, saponins, and polysaccharides in XXD.

### 4.3 Ge-gen-Qin-lian decoction

Ge-gen-Qin-lian decoction (GGQLD), a well-known TCM prescription for diabetes, is made up of Pueraria, Huangqin, Huanglian, and licorice. GGQLD treatment altered the overall gut microbiota structure and enriched many butyrate-producing bacteria (e.g., *Roseburia* and *Faecalibacterium*), lowering glucose and serum proinflammatory cytokine concentrations and attenuating intestinal inflammation. Treatment with GGQLD significantly increased the levels of SCFAs in rat feces. Furthermore, after treatment, the expression of immune-related genes such as Ifnrg1, Stat1, and Nfkb1 in pancreatic islets was significantly reduced. A study found that by interacting with intestinal bacteria, GGQLD significantly reduced FBG, glycosylated hemoglobin, and glycosylated serum protein levels in diabetic rats, as well as fasting serum insulin levels (56).The mechanisms of action of GGQLD might be related to the augmentation of the upregulation of the mRNA expression of adiponectin and adiponectin protein concentration (57). Saponins, flavonoids, and berberine were detected in GGQLD, and the efficacy of GQD might be attributed primarily to its key ingredient, berberine, which is likely to alleviate T2DM *via* the modulation of the intestinal microbiota, thereby reducing systemic and local inflammation.

### 4.4 Pi-Dan-Jian-Qing decoction

Pi-Dan-Jian-Qing decoction (PDJQD), which contains Astragalus, Pseudostellaria heterophylla, Atractylodes, Potentilla discolor Bunge, Scrophularia, Coptidis, Scutellaria, Pueraria, and Salvia miltiorrhiza Bunge, among other ingredients, has been used in clinic to treat T2DM (58). By increasing the relative abundances of *Akkermansia*, *Bacteroides*, *Blautia*, *Desulfovibrio*, and *Lactobacillus* while decreasing the relative abundance of Prevotella, PDJQD could reduce the F/B ratio. The modulatory effects of PDJQD on the TCA cycle, histidine metabolism, and tryptophan metabolism have been linked to changes in the abundance of *Akkermansia*, *Bacteroides*, and *Lactobacillus* (59). Treatment with PDJQD improved hyperglycemia, hyperlipidemia, IR, and pathological changes in the liver, pancreas, kidney, and colon in T2DM rats. The polysaccharide and flavonoid components of PDJQD may have reduced proinflammatory cytokine levels and inhibited oxidative stress by acting on the intestinal microbiota.

The mechanism of Chinese medicine prescription acting on intestinal microbiota in the treatment of diabetes is shown in **Table 2**.

**Table 2:** Progress on the prescription of Chinese herbal compounding acting on intestinal microbiota in the treatment of diabetes.

Chinese medicinesprescription	Effects on gut microbiota		metabolites		Therapeutic effect	Document
	Promotes	Reduces	Promotes	Reduces		
**Huang-Lian-Jie-Du Decoction**	*Akkermansia* *Blautia Parabacteroides*	*Aerococcus Corynebacterium Staphylococcus*	SCFAs Bile acid	Glucose TG,TC LDL HDL	Improves the metabolic disturbance of lipids, hyperglycemia, and inflammation and shapes the microbiome	24368167 30349514
**Xie-xin Decoction**	*Alloprevotella Adlercreutzia* *Blautia* *Barnesiella Papillibacter Lachnospiraceae*	*Coriobacteriaceae*	SCFAs	Glucose TG,TC LDL HDL	Significantly ameliorates hyperglycemia, lipid metabolism, and inflammation	29487347
**Ge-gen-Qin-lian Decoction**	*Faecalibacterium* *Roseburia* *Clostridium Ruminococcus* *Dorea Butyricicoccus Coprococcus*	*−*	SCFAs	Glucose	Reduces blood glucose levels, regulates intestinal microbiota, induces ileal gene expression, and relieves systemic and local inflammation	23219338 33359679
**Pi-Dan-Jian-Qing Decoction**	*Lactobacillus* *Blautia* *Bacteroides, Desulfovibrio* *Akkermansia*	*Prevotella*	SOD GSH-Px HDL	Glucose TG,TC LDL	Improves hyperglycemia; hyperlipidemia; insulin resistance (IR); and pathological changes of liver, pancreas, kidney, and colon	34938667

## 5 Effects of prepared Chinese medicine on intestinal microbiota and diabetes

Prepared Chinese medicine is made from Chinese herbal medicine as raw materials and is processed into specific dosage forms of Chinese medicine products according to the prescribed prescription and preparation process for the purpose of disease prevention and treatment. It has the properties of a stable nature, precise efficacy, and relatively small toxic side effects and is simple to take, carry, store, and keep. The following are the main effects and mechanisms of prepared Chinese medicine, which is commonly used in the treatment of diabetes.

### 5.1 Shen-Ling-Bai-Zhu powder

Shen-Ling-Bai-Zhu powder (SLBZP) is a TCM formulation that has been widely used to improve T2DM. SLBZP is composed of ginseng, schisandra, astragalus, yam, dioscorea, raspberry, maitake, poria, etc. It is rich in saponins, flavonoids and polysaccharides, which are its most important active ingredients. A study showed that, after high-dose SLBZP treatment, the relative abundance of *Roseburia*, *Lactobacillus*, *Staphylococcus*, and *Psychrobacter* significantly decreased, while the relative abundance of *Acinetobacter*, *Ochrobactrum*, *Prevotella*, *Anaerostipes*, *Bilophila*, and *Turicibacter* increased significantly in the 9-week rats (60). Changes in intestinal microbiota are followed by changes in their metabolites, such as SCFA levels. Furthermore, SLBZP significantly reduced insulin, IR, and leptin resistance in rats. SLBZP could alleviate chronic inflammation in rats based on changes in the serum levels of monocyte chemoattractant protein-1 (MCP-1) and interleukin 1 (IL-1) (61). The findings show that SLBZP can lower blood glucose, body weight, glycosylated hemoglobin, and lipid levels, allowing it to control obesity, relieve chronic inflammation, regulate intestinal microbiota and metabolites, and prevent T2DM.

### 5.2 Shen-Qi compound

Shen-Qi compound (SQC), composed of astragalus, ginseng, Lycium, etc., is a kind of TCM formulation that has been widely used to improve T2DM. Studies have demonstrated that SQC can reduce glycemic variability, alleviate the inflammatory response, etc. Astragalus, ginseng, and Lycium can provide polysaccharides and saponins, and the mechanism by which SQC treats diabetes by interacting with intestinal microbiota may be related to these two active ingredients. SQC intervention could regulate the serum levels of insulin and glucagon and improve injury to the intestinal mucosal barrier of GK rats. After SQC intervention, the ratio of Bacteroidetes to Firmicutes could be improved in the gut. Moreover, SQC improves glycolysis, gluconeogenesis, the citrate cycle, lipid metabolism, amino acid metabolism, and SCFA metabolism by regulating the relative abundance of *Blautia, Prevotellaceae, Rothia, Roseburia, Lactobacillus*, *Butyricimonas*, and *Bacteroides* (62).

### 5.3 Liu-Wei-Di-Huang pills

Liu-Wei-Di-Huang (LWDH) pills are a Yin-nourishing and kidney-tonifying prescription in TCM with promising pharmacological characteristics. Its main components are processed Rehmanniae Radix, Moutan Cortex, yam, Poria cocos, and Rhizoma Alismatis (63). LWDH Pills were reported to possibly be used to treat diabetes, that is, new applications of classic herbal formulae. The modulation of the gut microbiota by the flavonoids, saponins, and polysaccharides of LWDH is one possible mechanism for its diabetes treatment. GK rats treated with LWDH altered the microbial structure and promoted the abundance of bacteria in *Firmicutes*, including *Allobaculum*, *Lactobacillus*, and *Ruminococcus*’ increased SCFA levels involving butyric acid, propionic acid, and acetic acid. LWDH reduces T2DM and jejunal injury *via* intestinal bacterial action, with the SCFAs-GPR43/41-GLP-1 pathway being one possible mechanism (64).

### 5.4 San-Huang-Yi-Shen capsule

San-Huang-Yi-Shen capsule (SHYS) has been used in the treatment of diabetic nephropathy (DN) in the clinic for many years. SHYS is composed of ginseng, astragalus, angelica, donkey-hide gelatin, rhizoma, and other ingredients. The active ingredients flavonoids, saponins, and polysaccharides of SHYS may regulate the intestinal microbiota. SHYS affected the beta diversity of the gut microbiota community in DN model rats. SHYS could decrease the F/B ratio. SHYX treatment affected the relative abundances of Anaerovibrio, Allobaculum, Bacteroides, Lactobacillus, etc. SHYX regulates arginine biosynthesis, the TCA cycle, tyrosine metabolism, and arginine and proline metabolism in DN model rats by influencing intestinal microbiota and metabolite levels (65). SHYS treatment alters intestinal microbiota and metabolism, which modulates body weight, hyperglycemia, proteinuria, and renal pathology in DN rats.

### 5.5 Tang-Nai-Kang

Tang-Nai-Kang (TNK) is a kind of TCM that is a mixture of extracts from five herbal plants: Spica Prunellae Vulgaris, Fructus Ligustri Lucidi, Psidium guajava, Radix Ginseng, and Saururus Chinensis. Over the years, TNK has been widely used to treat diabetes mellitus. Studies have shown that TNK ameliorates glucose intolerance and IR in prediabetic SHR/cp rats and obese Zucker rats (66). TNK could alleviate hyperglycemia and improve the composition and abundance of the gut microbiota in diabetic KKAy mice. TNK treatment increased the abundance and diversity of intestinal microbial species, such as *Akkermansia* and *Allobaculum*, while decreasing *Lactobacillus* (67). The high-dose TNK treatment significantly reduced FPG levels while increasing body weight in KKAy mice (67). Other studies have shown that TNK treatment significantly decreased fasting serum insulin (FINS) and FBG; increased the insulin sensitivity index (ISI); improved impaired glucose tolerance; reduced the serum levels of interleukin-6 (IL-6), C-reactive protein (CRP), and tumor necrosis factor-Δ (TNF-Δ); and increased serum adiponectin in SHR rats (68). The underlying hypoglycemic mechanisms of TNK may be due to the high number of saponins it contains.

The mechanism of Chinese medicinal preparations on intestinal microbiota and diabetes mellitus is shown in **Table 3**.

**Table 3:** Research progress of Chinese medicine preparations acting on intestinal microbiota in the treatment of diabetes mellitus.

Prepared Chinese medicines	Effects on gut microbiota		Metabolites		Therapeutic effect	Document
	Promotes	Reduces	Promotes	Reduces		
**Shen-ling-Bai-zhu Powder**	*Psychrobacter* *Lactobacillus* *Roseburia* *Staphylococcus*	*Anaerostipes* *Turicibacter* *Bilophila* *Ochrobactrum* *Acinetobacter* *Prevotella*	SCFAs insulin	Glucose TG,TC LDL HDL	Regulates intestinal microbiota and metabolites and relieves chronic inflammation to control obesity	35285199
**Shen-Qi Compound**	*Bacteroidetes*	*Firmicutes*	SCFAs insulin	Glucose TG,TC	Improves gluconeogenesis, glycolysis, amino acid metabolism, lipid metabolism, the citrate cycle, and butanoate metabolism	35219959
**Liu-Wei-Di-Huang Pills**	*Allobaculum Lactobacillus Ruminococcus*	*−*	SCFAs insulin	Glucose	Reduces blood glucose levels and regulates intestinal microbiota	35857109
**San-Huang-Yi** **-Shen Capsule**	*Lactobacillus* *Allobaculum Anaerovibrio* *Bacteroides*	*Candidatus_Sacchari-monas*	L-Glutamine Acetylcholine	L-Phenylal-anine L-Tyrosine	Alleviates hyperglycemia and improves renal function, pathological changes in the kidney, oxidative stress, and inflammatory response	35058786
**Tang-Nai-Kang**	*Akkermansia Allobaculum*	*Lactobacillus*	FPG insulin	CRP Glucose	Reduces blood glucose levels and increases the insulin sensitivity index	34868327 31682379

## 6 Summary and prospect

Beneficial Qi TCM promotes the growth of probiotics, inhibits the colonization of pathogenic bacteria in the intestine, and influences intestinal epithelial cell differentiation and apoptosis, and positive regulation of the gut microbiota is beneficial in the treatment of metabolic syndromes. Several studies have shown that the beneficial role of TCM is related to gut microbiota regulation. Chinese medicine can modulate the composition and activity of the metabolites of intestinal bacteria by affecting their growth. For example, herbal medicine makes the metabolites of the intestinal microbiota increase in SCFAs, which can regulate the composition and activity of their metabolites by influencing growth, regulating the concentration of metabolites, disrupting the intestinal barrier, improving insulin sensitivity, regulating lipid metabolism and blood glucose levels, and improving the level of inflammation, which can explain why TCM plays a role in a variety of metabolic diseases such as diabetes (69).

TCM can regulate the composition of intestinal microbiota and its metabolites, and intestinal microbiota can also transform and promote the absorption of Chinese herbal ingredients. This paper discusses the role of gut microbes in the development of T2DM, as well as how to treat T2DM by targeting the gut microbiota with herbs and their active ingredients (70). The active ingredients such as saponins, flavonoids, polysaccharides, and alkaloids contained in TCM can act on intestinal microbiota, which is a possible mechanism to treat diabetes. Changes in the abundance of certain pathological bacteria contribute directly to the development of diabetes. By utilizing intestinal mucin, intestinal probiotics can mediate the metabolism of TCM ingredients and *in vivo* substances, as well as maintain the integrity of the gut barrier, such as *Akkermansia muciniphila*. It may be a promising method of controlling T2DM because it can relieve T2DM through various mechanisms and modes of action, such as improving microbial metabolism and protecting the intestinal barrier function and having an anti-inflammatory effect (71, 72). All of these help us understand current TCM research and development and provides a foundation for future clinical applications (73).

However, there is still a long way to go in explaining the mechanism of TCM against metabolic diseases and its potential side effects, particularly given the wide variations in the composition of intestinal microbiota among individuals. We should continue to improve our understanding of the common signal transduction mechanism in different bacteria in order to achieve standardized treatment by targeting common molecules or signaling pathways, with the ultimate goal of translating knowledge into practice in mind.

## Data availability statement

The original contributions presented in the study are included in the article/supplementary material. Further inquiries can be directed to the corresponding author.

## Author contributions

MS and CH conceived the paper. MS, CH, and RH analyzed the relevance of the literature and wrote the article. TT and WT revised the figures and reviewed the article. All authors reviewed and approved the final version of the manuscript.
